# Therapeutic potential of green-synthesized silver nanoparticles: Combating biofilms of multidrug-resistant *Staphylococcus aureus* RM-Ph8 and modulating the immune response in the liver tissue of rats

**DOI:** 10.14202/vetworld.2024.2211-2224

**Published:** 2024-10-04

**Authors:** Mohamed T. Shaaban, Sahar H. Orabi, Marwa Salah Abdel-Hamid, Reda M. S. Korany, Fatimah M. Alshehrei, Rania Hamed Elbawab

**Affiliations:** 1 Department of Botany and Microbiology, Faculty of Science, Menoufia University, Egypt; 2 Department of Biochemistry and Chemistry of Nutrition, Faculty of Veterinary Medicine, University of Sadat City, Sadat, Egypt; 3 Department of Microbial Biotechnology, Genetic Engineering and Biotechnology Research Institute, University of Sadat City, Egypt; 4 Department of Pathology, Faculty of Veterinary Medicine, Cairo University, Giza, Egypt; 5 Department of Biology, Jumum College University, Umm Al-Qura University, P.O Box 7388, Makkah, 21955, Saudi Arabia

**Keywords:** *Artemisia annua* bactericidal activity, immune histochemical expression, multidrug-resistant *Staphylococcus aureus*, phyto-AgNPs

## Abstract

**Background and Aim:**

The emergence of multidrug-resistant *Staphylococcus aureus* (MRSA) strains poses a significant threat to healthcare settings. Although various studies have explored alternative antibiotics, discovering novel therapeutic agents remains crucial. This study aimed to synthesize green silver nanoparticles (AgNPs) as bactericidal agents, identify a multidrug-resistant isolate of *Staphylococcus aureus*, and explore their biofilm formation ability. To estimate the role of phyto-AgNPs in the perfection of immune markers and healing hepatic lesions *in vivo*.

**Materials and Methods:**

The clinical isolate of MRSA was identified using 16S rRNA New green AgNPs derived from *Artemisia annua* extract were synthesized. The nanoparticles (NPs) were characterized, and their minimum inhibitory concentration was estimated for fighting MRSA biofilm. A study was conducted on rats to evaluate the effect of new NPs on their immune response to MRSA infection.

**Results:**

The new clinical isolate of MRSA RM-Ph8 was identified by molecular phylogenetic analysis as *S. aureus*, and 16S rRNA sequence analysis confirmed that the new strain was similar to *S. aureus* with 98.12% identity with accession number OQ421819. The FTIR of the new phyto-AgNPs displayed different functional groups that work as reducing silver nitrate agents. Transmission electron microscopy and scanning electron microscopy images showed spherical particles with an average diameter of 6–28 nm smaller. The chemical method led to complete cell destruction of the multidrug strain within 24 h. Biofilm formation showed that the new MRSA clinical strain was strongly adherent (88%). Notably, the phyto-AgNPs exhibited significant bactericidal activity against the new MRSA strain, with an MIC of up to 50 mg/mL. Moreover, phyto-AgNPs significantly decreased reversed MRSA-induced liver and kidney function impairment, with improvement in both the histopathological lesions and immune histochemical expression of tumor necrosis factor-α and inducible nitric oxide synthase at p < 0.05 compared with the untreated group.

**Conclusion:**

Green AgNPs are a promising therapeutic approach against multidrug-resistant bacterial infections, surpassing the effectiveness of conventional antibiotics.

## Introduction

The development of silver nanoparticles (AgNPs) offers a promising alternative for controlling multidrug-resistant (MDR) microorganisms. Bacteria are less prone to developing resistance to conventional antimicrobial agents against metal nanoparticles (NPs) than traditional antibiotics [1]. This approach can be used to treat various bacterial infections and facilitate the diagnosis of chronic diseases [2]. Consequently, multidrug-resistant *Staphylococcus aureus* (MRSA) is a major opportunistic pathogen responsible for a wide range of infections resistant to antibiotics and forming biofilms, causing considerable problems in medicine and public health [3, 4]. MRSA can cause infections in different organs and tissues of humans and other animals; including skin wound infections, folliculitis, pneumonia, endocarditis, and bacteremia [5, 6]. AgNPs have bactericidal effects that rely on the specific binding of microorganisms to the surface of antimicrobial agents, which inhibits the physiological activity of the bacteria and kills cells [7, 8]. Furthermore, previous study has demonstrated the effectiveness of NPs in the delivery of therapeutic drugs [9].

The green synthesis of AgNPs offers eco-friendly and biocompatible alternatives that preserve their antimicrobial properties while addressing potential health risks to humans, animals, and the environment [10, 11]. Natural products such as clove (*Syzygium aromaticum*) inhibit the activity of various MDR bacteria [12], and cinnamon can control urinary tract infections caused by extended-spectrum beta-lactamase-producing *Escherichia coli* [13]. In addition, thyme (*Thymus vulgaris*) and jujube (*Ziziphus jujuba*) have antibacterial activities against MRSA strains [14]. These natural products contain phytochemical elements that substantially influence the stability, activity, and physical characteristics of NPs [15, 16]. In particular, *Artemisia annua* contains multiple bioactive compounds, such as phenols and terpenoids that provide antioxidant and anti-inflammatory properties. These properties are beneficial for the treatment of MRSA infections in the lung tissue of rats [17]. The antibacterial activity of *A. annua* essential oil has been tested against *S. aureus*, *Pseudomonas aeruginosa*, and *E. coli* and exhibited considerable bactericidal activity [18]. Green processes in NP biogenesis are simpler, non-toxic, and more economical than chemical methods, and they have demonstrated antiparasitic effects [19, 20].

This investigation focused on the identification of the new MRSA clinical isolate RM-Ph8 and confirmed the synthesis of phyto-AgNPs from *A. annua* as a bactericidal agent. In addition, this study explored the ability of these NPs to produce biofilms and evaluated their role in improving immune markers and healing hepatic lesions *in vivo*.

## Materials and Methods

### Ethical approval

The research protocol (VUSC-012-1-22) was approved by the Ethics Committee on Animal Care and Use at the Faculty of Veterinary Medicine, University of Sadat City, Egypt. All experiments were conducted in accordance with institutional guidelines for animal research and the ARRIVE guidelines.

### Study period and location

The study was conducted from June 2022 to September 2023. All works were done at the Microbial Biotechnology Department, Genetic Engineering and Biotechnology Research Institute, University of Sadat, in cooperation with the Biochemistry and Chemistry of Nutrition Department, Faculty of Veterinary Medicine, University of Sadat, Sadat, Egypt.

### NP synthesis and physiochemical properties

*A. annua* leaves were bought from the local Harraz market, Cairo, Egypt. An ethanol extract (96%) of *A. annua* was used to prepare 100-μM silver nitrate solution [18]. *A. annua* extract (10 mL) was added dropwise to the silver nitrate solution separately at 80°C until the solution changed to a brownish-yellow color in <60 min, indicating that the silver ions had undergone bio-reduction. Fourier transform infrared spectroscopy (FTIR) spectra were recorded at a resolution of 4 cm^−1^ using an FTIR spectrometer (8000 series) with KBr in the wavenumber range of 4000–400 cm^−1^. Scanning electron microscopy (SEM) (Jeol JSM-6100, Tokyo, Japan) was used to evaluate NP size, surface morphology, and dispersion. Transmission electron microscopy (TEM) (Jeol 1200-EXII) was used to determine the size and shape of the new phyto-AgNPs, as well as the antibacterial effect of green AgNPs on MRSA cell structure. The specimens intended for TEM analysis were rinsed with phosphate-buffered saline (PBS) and immersed in a fixative solution containing 4% glutaraldehyde in sodium cacodylate buffer at 4°C for 3 h. Thereafter, specimens were serially dehydrated by exposure to increasing ethanol concentrations. After dehydration, the specimens were subjected to critical point drying using a BAL-TEC CPD 030 (Liechtenstein, Tokyo, Japan) and sputter-coated with gold using a BAL-TEC SCD 005 (Liechtenstein) at 100 kV.

### Molecular identification of a novel MDR S. *aureus* clinical isolate

The clinical isolate of MRSA RM-Ph8 was obtained from patients in the intensive care unit of Menoufyia University, Egypt [18]. This new clinical isolate was identified at the molecular level using the 16S rRNA gene, as described by Hashem *et al*. [21]. Total DNA was extracted from frozen bacterial samples containing 1–2 × 10^8^ cells, which were thawed and brought to room temperature (15°C–25°C) before starting the procedure. Genomic DNA was extracted using a genomic DNA extraction kit (Intron Biotechnology, Korea) and visualized on 1% agarose gels [22]. The 16S rRNA gene of the MRSA RM-Ph8 isolate was amplified using a thermocycler (Biometra thermocycler, Germany) with universal primers: Bact 27F (5′-AGAGTTTGATCACTGGCTCAG-3′) and Bact 1492R (5′-TACGGCTACCTTGTTACGA CTT-3′) [23, 24].

A precise protocol was followed during the polymerase chain reaction (PCR) amplification procedure, which started with an initial denaturation step at 94°C for 5 min, followed by 35 cycles of denaturation at 94°C for 30 s, annealing at 57°C for 30 s, and elongation at 72°C for 1 min. The final elongation phase lasted for 7 min at 72°C. An ABI 3730xl DNA sequencer was used for PCR product sequencing by combining traditional Sanger technology with 454 sequencing to reduce data gaps and ambiguities and effectively reduce the project duration. Furthermore, the nucleotide sequences of the 16S rRNA gene were registered in the NCBI database using BLAST (http://www.ncbi.nlm.nih.gov/blast/). MEGA-integrated software for molecular evolutionary analysis (http://www.megasoftware.net/) was used for the phylogenetic analysis. Using multiple alignments (http://multalin.toulouse.inra.fr/multalin/), the nucleotide sequence differences among the studied samples were identified [25].

### Determination of minimum inhibitory concentration (MIC) for screening the antibacterial activity of green-synthesized AgNPs

The efficacy of both phyto-and chemically synthesized AgNPs was assessed against new MRSA RM-Ph8, which was incubated with the three treatments at various dilutions: 10, 30, 50, 90, and 120 mg/mL. 100 μL of fresh culture (0.1 optical density [OD] value; 600 nm) was inoculated into the tubes for 24 h at 37°C, and the turbidity of the bacterial growth was determined using a spectrophotometer at an OD of 600 nm. The lowest dilution that completely inhibited visible bacterial growth was considered the MIC [26].

### Biofilm potential of the new MRSA strain against phyto-AgNPs

The ability of MRSA RM-Ph8 to form biofilms was assessed *in vitro* by observing the binding of crystal violet dye to adherent cells [27]. The potential of phyto-AgNPs to prevent biofilm formation in RM-Ph8 was also investigated. The growth medium was supplemented with the specified components during inoculation to allow the cells to develop a biofilm. Consecutive two-fold dilutions of phyto-AgNPs were created in a 96-well microtiter plate (MTP) containing trypticase soy broth containing 1% glucose (TSBGlc, Merck KGaA, Darmstadt, Germany) at different concentrations (0.5, 0.25, 0.125, 0.0625, 0.031, 0.016, and 0.008 mg/mL). Bacterial suspensions (final concentration of 5 × 10^8^ CFU/mL per well) were then added to the MTP. After incubation at 37°C for 24 h, the effect of phyto-AgNPs on the growth of MRSA RM-Ph8 was measured using a microplate reader (Statfax, USA) at an OD of 620 nm.

Biofilm formation in the presence of AgNPs was evaluated by first discarding the bacterial cells and washing the wells 3 times with PBS (pH 7.2). The wells were dried for 30 min and then fixed with 99% methanol for 10 min. Subsequently, each well was treated with 0.1% crystal violet and stained for 20 min. Excess crystal violet was discarded, and the wells were washed with distilled water. After drying the wells, 200 μL of 30% acetic acid was added. Biofilm formation in the tested wells was compared with that in the untreated controls, and the percentage reduction was calculated. All tests were performed in triplicate, and the results were reported as the average of the three replicates [28]. The average of the three ODs was determined, and the standard deviation was calculated. The results were categorized as follows: <17%, negative; 17%–34%, weakly positive; 35%–67%, moderately positive; and >68%, strongly positive [29].

### Effects of green-synthesized AgNPs against RM-Ph8-induced MRSA infection

For this objective, 36 male albino rats (150 g) were acclimatized for 7 days under hygienic conditions, provided with a standard basal diet (containing corn, soybeans, essential minerals [calcium, phosphorus, and sodium], baking soda, vitamins, and a binding agent [lecithin]) [30], and maintained under a controlled environment. The rats were divided into six groups of six rats per group (Table-1**)**. The rats were anesthetized after 2 weeks, and blood samples were collected using heparinized capillary tubes. Clear serum was stored for biochemical analysis, and liver samples were excised and rinsed with 0.95% saline.

**Table-1:** Experimental Design and treatments of albino rats experiment.

Group	Treatment	Concentration dose/duration time
A (control)	Rats received normal saline	(100µl) orally/14 days
B	Rats were given biogenic AgNPs	50 mg/kg orally for 14 days
C	Rats were administered with *A. annua* extract	50 mg/kg orally for 14 days
D	Rats infected with *S. aureus*	0.2 ml s/c for 2 days
E	Rats were infected with *S. aureus* in then administered with *A. annua* ethanolic extract	0.2 ml s/c for 2 days 50 mg/kg orally for 14 days
F	Rats were infected with *S. aureus* in then administered with *A. annua* ethanolic extract	0.2 ml s/c for 2 days 50 mg/kg orally for 14 days

### Biochemical profiles

Serum aspartate aminotransferase (AST) activity, alanine aminotransferase (ALT) activity, creatinine levels, and urea levels were assayed in accordance with the manufacturer’s instructions. These kits were purchased from Spectrum Diagnostics (Egypt) to determine serum AST (Cat No. AS 1061[33]), ALT (Cat. No. AL 1031[33]), urea (Cat No. UrR 2110), and creatinine (Cat No. CR 1250). The serum concentrations of creatinine and urea, which serve as kidney function tests, were assessed using colorimetric methods based on procedures described by Bowers and Wong [31] for creatinine and Shephard and Mezzachi [32] for urea. Serum AST and ALT levels were determined using a previously described method [33]. Furthermore, we quantified serum C-reactive protein levels (CRP), an inflammation marker, using an enzyme immunoassay technique and a diagnostic kit as described by Helgeson *et al*. [34].

### Histopathological microscopic examination and lesion scoring

Liver tissue samples were collected from each group and preserved in 10% neutral-buffered formalin. The samples were then cleaned, dried, and embedded in paraffin. The paraffin-embedded blocks were sliced into 5 μm thick sections and stained with hematoxylin and eosin for histological examination. The stained sections were examined using a light microscope (Olympus BX50, Japan) [35, 36].

Histopathological alterations in liver tissue were noted and assigned a score based on the severity of the changes: No change (0), mild (1), moderate (2), and severe (3). Based on the percentage of changes observed, the following was used to determine the grading: <30%, mild change; 30%–50%, moderate change; and >50%, severe change [37, 38].

### Immunohistochemical staining of liver tissue

Liver tissue sections were deparaffinized using xylene and rehydrated with a series of graded alcohol concentrations. A hydrogen peroxide block (Thermo Scientific, USA) was applied to inhibit endogenous peroxidase activity. Antigen retrieval was performed by treating tissue sections with 10-mM citrate in a microwave oven for 10 min. The sections were treated with one of the following primary antibodies: rabbit anti-caspase-3 (diluted to 1:1000; Abcam, Cambridge, UK) or tumor necrosis factor-α (TNF-α) (diluted to 1:100; Celltech Ltd., UK) for 2 h. Subsequently, the sections were rinsed with PBS and incubated withgoat anti-rabbit immunoglobulin G H&L (HRP) (ab205718; Abcam, Cambridge, UK) for 10 min, followed by another rinse with PBS. Subsequently, the sections were treated with 3,3-diaminobenzidine tetrahydrochloride (DAB; Sigma, USA) and counterstained with hematoxylin before mounting. The primary antibodies were replaced with PBS as negative controls [39, 40].

### Quantitative assessment of TNF-α and inducible nitric oxide synthase (iNOS) immunoreactivity in liver tissue sections

We calculated the immune response of TNF-α and iNOS in liver tissue sections from each group [41]. Immunoreactivity was assessed in 10 microscopic fields per section under high-power microscopy (Olympus) at 400× magnification. The percentage of positively stained cells was determined using color deconvolution with ImageJ 1.52p software (Wayne Rasband, National Institutes of Health, USA).

### Statistical analysis

The data were statistically assessed using one-way analysis of variance and Duncan’s *post hoc* test to confirm the statistical differences among groups [42], with a p < 0.05 considered statistically significant.

## Results

### Phytosynthesis and characterization of AgNPs

**Figure-1:**
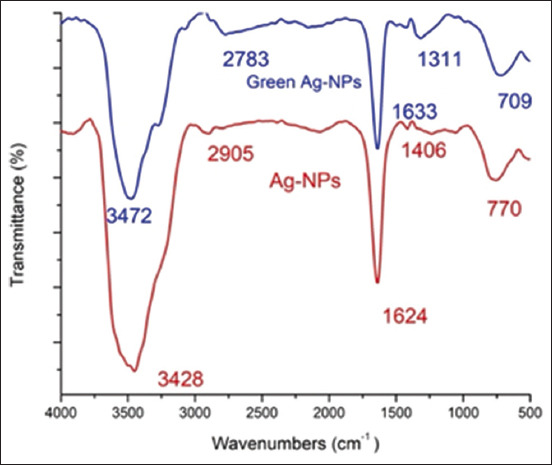
Fourier transform infrared spectra of the new green and chemical Sliver nanoparticles.

Figure-1 and Table-2 show the FTIR spectra of the chemical and phytosynthesis of AgNPs using *A. annua*, which exhibited five peaks, and the same peak positions were repeated for amine N-H stretch, amide C=O stretch, and aromatic CH bending in both NPs. Furthermore, the chemical AgNP synthesis showed two different functional groups: An alkane C-H stretch and O-H bending, which are the absorption peaks at 2905 and 1406 cm^−1^, respectively. AgNPs were prepared using *A. annua* in the FTIR spectrum and formed two peaks at 2777 and 1311 cm^−1^, which are aldehyde C-H stretch and –NO_2_ stretch, respectively.

**Table-2:** Fourier transform infrared spectroscopic (FTIR) peaks of phyto and Chemical Ag-NPs.

No.	Functional groups	Absorption (s) in literature (cm^1^)	Chemical AgNPs peak value	Phyto-AgNPs peak value
1	Amine N-H stretch	3550–3250	3428	3472
3	Alkanes C-H stretch	2990–2850	2905	---
4	Aldehydes C-H stretch	2800–2700	---	2777
6	Amide C=O Stretch The carbonyl stretch	1600–1650	1624	1633
7	Hydroxyl group O-H bending	1330–1430	1406	---
8	-NO_2_ stretch	1390–1300	---	1311
9	Aromatic -CH Bending	680–860	770	709

**Figure-2:**
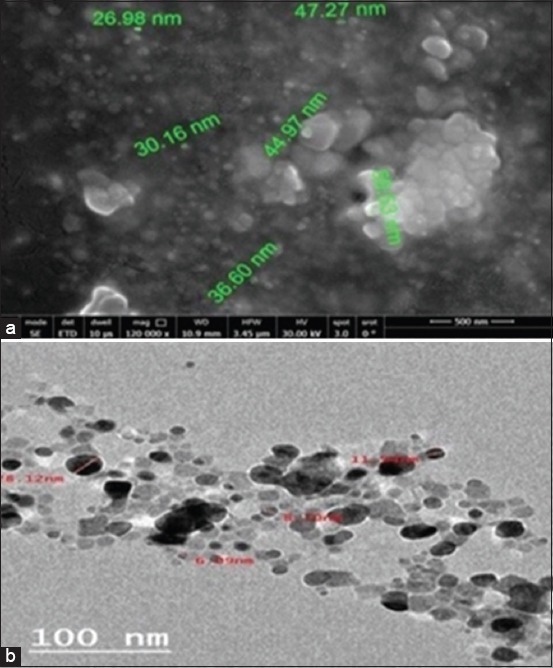
(a) Scanning electron microscopy 500 nm and (b) transmission electron microscopy 100 nm examination of green AgNPs (phyto-AgNPs). AgNPs=Sliver nanoparticles.

SEM images of the lyophilized phyto-AgNPs mostly showed white spherical particles smaller than 100 nm (Figure-2a). The TEM images indicated that the synthesis resulted in the formation of spherical, monodispersed AgNPs that were well distributed with minimal agglomeration (Figure-2b). The sizes of the phytosynthesized AgNPs ranged from 4 to 15 nm, smaller than AgNPs, which typically range from 8 to 46 nm.

### Molecular identification of a new strain via 16S rRNA gene sequencing

**Figure-3:**
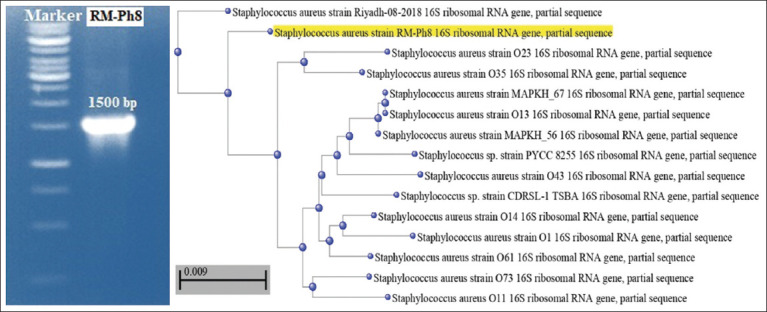
Phylogenetic tree with reference strains of *Staphylococcus aureus* and ethidium bromide-stained agarose gel resolving the Polymerase Chain Reaction amplification fragment of the 16S rRNA gene ~1500 Kbp of the new strain MRSA RM-Ph8. DNA marker (250–10000 bp).

PCR amplification of DNA samples from RM-Ph8 isolates successfully amplified a PCR product with an expected size of 1500 bp. Figure-3 shows agarose gel resolving of the PCR amplification of the 16S rRNA gene (~1500 bp fragment) from the clinical isolate, which was then identified by DNA sequencing as *S. aureus* strain RM-Ph8. The sequence was submitted to GenBank (accession number: OQ421819; *S. aureus* strain RM-Ph8 16S ribosomal RNA gene, partial sequence https://www.ncbi.nlm.nih.gov/nuccore/OQ421819). In addition, the phylogenetic tree shows a high genetic relationship (98.12%) with the reference strains of *S. aureus* (Figure-3).

### MIC of phytosynthesized AgNPs

The most sensitive MIC among the examined concentrations of phytosynthesized AgNPs was 50 mg/mL (Table-3), which reflects the bactericidal activity against the new MRSA RM-Ph8 strain within 24 h. Interestingly, the MIC values of both chemically synthesized AgNPs and *A. annua* extract exhibited significantly similar concentrations (90 mg/mL).

**Table-3:** Minimum inhibition concentration turbidity of green and chemical AgNPs on MR* Staphylococcus aureus* RM-Ph 8 strain.

Treatments	Control	10mg/ml	30mg/ml	50mg/ml	90mg/ml
Optical density of MR *S. aureus* RM-Ph 8 strain					
Biogenic AgNPs	0.51 ± 0.003^a^	0.35 ± 0.008^a^	0.27 ± 0.003^a^	0.13 ± 0.008^a^	0.20 ± 0.02^a^
Chemical AgNPs	0.51 ± 0.003^a^	0.40 ± 0.017^b^	0.40 ± 0.003^c^	0.35 ± 0.003^b^	0.32 ± 0.003^b^
*Artemisia annua* extract	0.51 ± 0.003^a^	0.55 ± 0.006^c^	0.52 ± 0.012^b^	0.49 ± 0.05^c^	0.49 ± 0.001^b^

### Tissue culture microtiter

**Figure-4:**
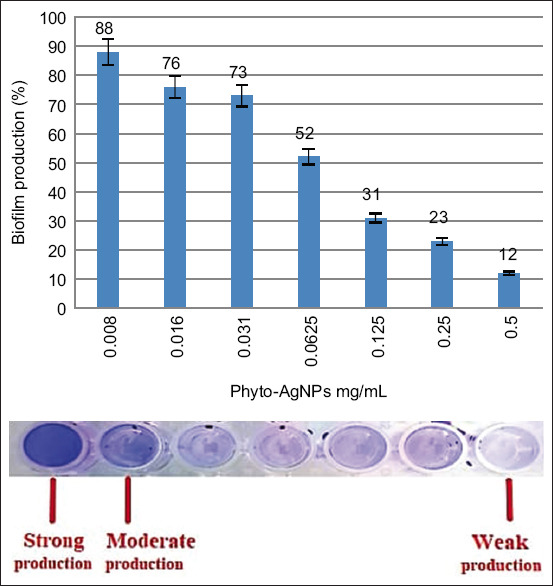
Percentage of biofilm adherence ability of new MRSA RM-Ph8 strain, microtiter plate demonstrating the power of adherence to weak, moderate, and strong conditions. MRSA=Multidrug-resistant *Staphylococcus aureus*.

MRSA RM-Ph8 cells were cultured to develop biofilms over 24 h in MTPs. Subsequently, the biofilms were subjected to different concentrations of AgNPs (0.5, 0.25, 0.125, 0.0625, 0.031, 0.016, and 0.008 mg/mL) (Figure-4). These MTPs demonstrated the moderate effect of phyto-AgNP dilution (0.25–0.008 mg/mL) on biofilm formation.

The ability of MRSA RM-Ph8 to form a biofilm was detected using a crystal violet assay. The biofilm adherence ability percentage of the new MRSA RM-Ph8 strain in the presence of phyto-AgNP dilutions (0.5, 0.25, 0.125, 0.0625, 0.031, 0.016, and 0.008 mg/mL) was determined, which provided adherence ability percentages (12, 23, 31, 52, 73, 76, and 88%, respectively) (Figure-4). Thus, the 0.008 mg/mL dilution exhibited the highest percentage of biofilm formation (strongly adherent), whereas higher phyto-AgNP concentrations significantly reduced biofilm formation. This is clearly shown in Figure-4, where treatment with 0.5 mg/mL of these NPs nearly prevented the formation of biofilms (weakly adherent).

**Figure-5:**
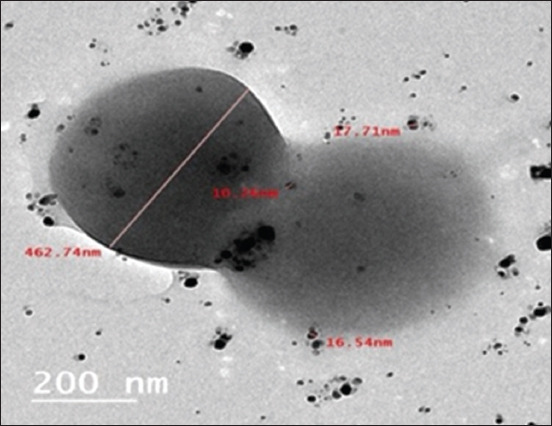
TEM examination of the new MRSA RM-Ph8 strain treated with AgNPs based on *Artemisia annua* extract at a 200-nm scale bar. The antibacterial activity of phyto-AgNPs and chemical AgNPs against weak, moderate, and strong adhesion was demonstrated. AgNPs=Sliver nanoparticles. MRSA=Multidrug-resistant *Staphylococcus aureus*.

The TEM image of the new MRSA RM-Ph8 strain after treatment with AgNPs prepared from *A. annua* extract indicated that the synthesized AgNPs were spherical and capped with plant compounds, which were all <100 nm in diameter (Figure-5). After 24 h of exposure, the new MRSA RM-Ph8 strain was completely lysed and destroyed. The treated cells lost their cellular functions, and all that remained was a dehydrated mass of cellular components and debris. Figure-5 shows the antibacterial effects of the phytosynthesized and chemically synthesized AgNPs. The phyto-AgNPs were significantly more effective against the new MRSA RM-Ph8 strain *in vitro* than the chemical AgNPs.

### Biochemical profiles

**Figure-6:**
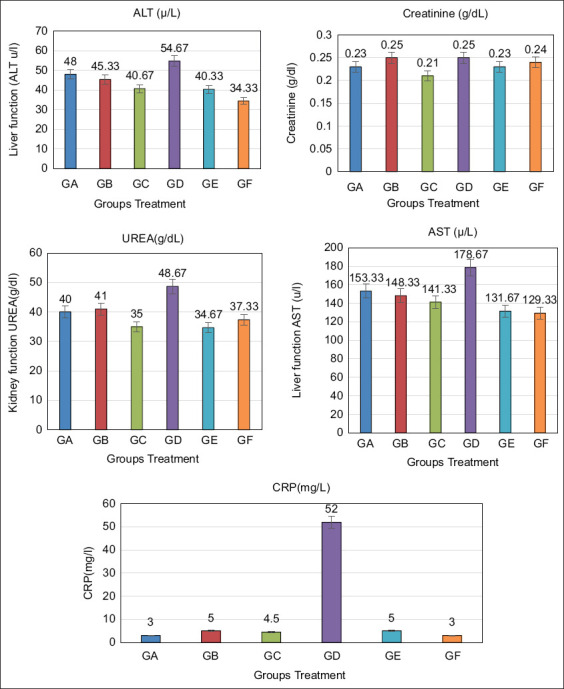
Effect of extract of *Artemisia Annua*, phyto-AgNPs, and/or MRSA RM-Ph8 strain on liver and kidney function biomarkers in rats. Values are expressed as means ± SE (standard error). The mean difference is significant at p < 0.05. Data with different letters in the same row are significantly different. GA: (control group), GB: (*A. annua* AgNPs group), GC: (*A. annua* extract group), GD: (MRSA infected group), GE: (MRSA and *A. annua* extract group), GF: MRSA infected and phyto *-*sliver nanoparticles group). AgNPs=Sliver nanoparticles. MRSA=Multidrug-resistant *Staphylococcus aureus*.

Figure-6 shows increased ALT, AST, urea, and CRP levels in the *S. aureus*-infected group compared with the control group, and the comparison between the infected and control groups did not reveal any notable variance in creatinine levels. Nevertheless, administering *A. annua* and biogenic AgNPs in infected rats normalized ALT, AST, CRP, and urea levels.

### Analysis of microscopic examination of liver issues

**Figure-7:**
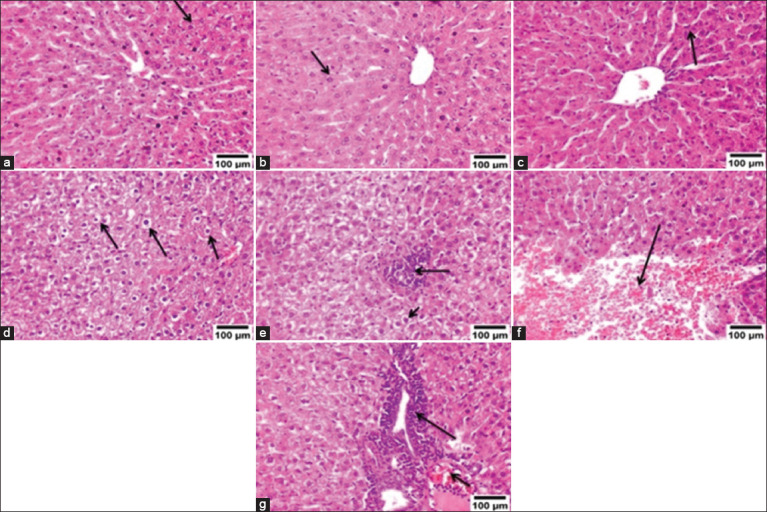
Photomicrograph of rat liver. (a–c) show normal histological structure of hepatocytes (arrow) for Groups A, B, and C, respectively. (d) Group D shows vacuolar degeneration and necrosis of hepatocytes (arrows). (e) Group D shows the area of focal necrosis replaced by inflammatory cell aggregation (arrow). (f) Group D shows a large area of hemorrhage (arrow). (g) Group D shows portal mononuclear inflammatory cell infiltration (long arrow) and congestion of portal blood vessels (short arrow) (H&E 200×).

**Figure-8:**
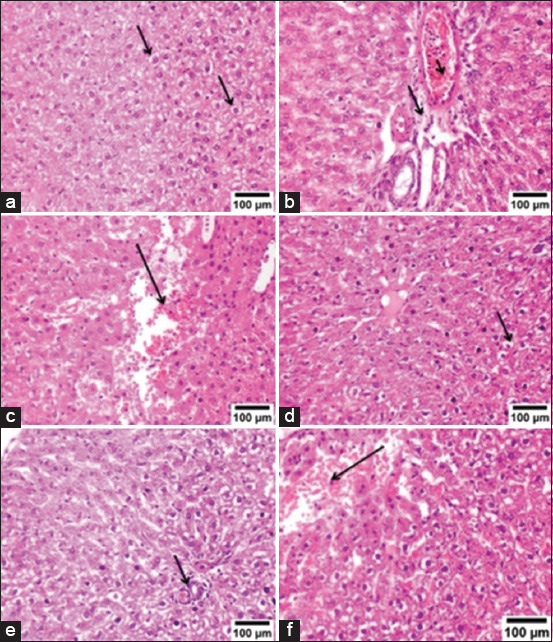
Photomicrograph, rat liver. (a) Group E shows mild vacuolar degeneration of hepatocytes (arrows). (b) Group E shows few mononuclear inflammatory cell infiltrations in the portal area (long arrow) and congestion of portal blood vessels (short arrow). (c) Group E shows the area of hemorrhage between hepatocytes (arrow). (d) Group F shows mild vacuolar degeneration in a few hepatocytes (arrow). (e) Group F shows a normal portal area (arrow). (f) Group F shows a small area of hemorrhage (arrow) (H&E 200×).

Liver samples from groups A, B, and C exhibited normal histological structures (Figures-7a–c). Group D showed vacuolar degeneration and necrosis in numerous hepatocytes (Figure-7d), with areas of focal necrosis replaced by inflammatory cell aggregation (Figure-7e). There were also large areas of hemorrhage between the hepatocytes (Figure-7f), and portal areas revealed mononuclear inflammatory cell infiltration with congestion of the portal blood vessels (Figure-7g). In Group E, the liver showed mild vacuolar degeneration in some hepatocytes (Figure-8a), infiltration with few mononuclear inflammatory cells in the portal areas, and congestion of the portal blood vessels (Figure-8b), with the presence of hemorrhagic areas between the hepatocytes (Figure-8c). Group F showed mild vacuolar degeneration in a few hepatocytes (Figure-8d), nearly normal portal areas (Figure-8e), and small areas of hemorrhage (Figure-8f). Liver lesions were assessed and scored according to severity (Table-4).

**Table-4:** Histopathological lesions scoring in liver of all groups.

Lesions	Group A	Group B	Group C	Group D	Group E	Group F
Vacuolar degeneration of hepatocytes	0	0	0	3	2	1
Focal necrosis with inflammatory cells infiltration	0	0	0	1	0	0
Areas of hemorrhage	0	0	0	3	2	1
Inflammatory cells infiltration in portal areas	0	0	0	2	1	0
Congestion of portal blood vessels	0	0	0	3	2	1

The score system was designed as: score 0=absence of the lesion in all rats of the group (n = 6), score 1= (<30%), score 2= (<30% – 50%), score 3= (>50%) .GA: (control group), GB: (*A. annua* nano silver group), GC: (*A. annua* extract group), GD: (MRSA infected group), GE: (MRSA and *A. annua* extract group), GF: MRSA infected and *A. annua* nano silver new group)

### Hepatic TNF-α and iNOS protein expression in liver tissue

**Figure-9:**
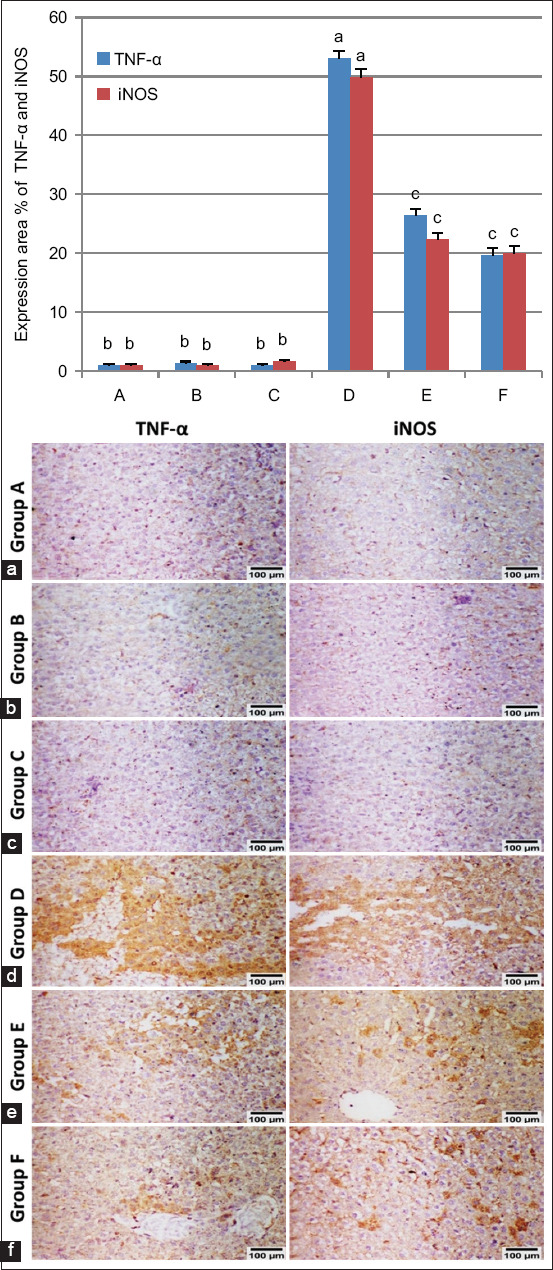
Immunostaining of TNF-α and iNOS in liver rats. (a–c) show very weak to no immune reactive cells for Groups A, B, and C, respectively. (d) Group D shows strong expression of both markers. (e and f) show moderate to weak positive immune reactions in a few cells (TNF-α and iNOS 200×) for E and F groups, respectively. (g) Immunostaining expression area % of TNF-α and iNOS (data expressed as mean ±SE, different letters (a–c) indicating significant differences at p < 0.05).

Immunostaining of the liver for TNF-α and iNOS indicated minimal to absent immunoreactive cells in Groups A, B, and C (Figures-9a–c). Group D showed strong expression of both markers (Figure-9d), and Groups E and F showed moderate to weak positive immunoreactivity in a few cells for TNF-α and iNOS (Figures-9e and f). Immunostaining findings showing TNF-α and iNOS expression as percentage areas in liver tissue from various groups are presented in Figure-9g.

## Discussion

Herbal plants are rich in bioactive compounds and useful for preparing NP to fight against antibiotic-resistant bacteria. Green synthesis of NPs has unique properties because NPs are safe and biocompatible and have promising applications in biomedicine and related fields [43]. In this study, we successfully synthesized green AgNPs using *A. annua* extract according to the methods described by Shaaban *et al*. [18], Massiha *et al*. [44], and Johnson *et al*. [45]. Furthermore, we confirmed the antiviral properties of *Artemisia* spp., which possess capping and therapeutic properties that are widely used for treating various diseases due to their efficacy.

The FTIR spectrum was measured over a wavelength range of 500–4000 cm^−1^. The slight variation in peak absorbance could be attributed to differences in particle size. Verma and Mehata [46] suggested that compounds present in herbal plant extracts, including flavonoids and terpenoids, could serve as reducing agents that facilitate the conversion of silver salts into AgNPs.

The FTIR spectra identified active groups in the molecules that stabilize and coat the newly synthesized AgNPs, which were prepared using a biological method. The analysis further revealed the differences resulting from the interaction between the functional groups of *Artemisia* and Ag^+^ ions during biosorption. The broad peak at 3428 cm^−1^ in the biomass sample before Ag^+^ biosorption was attributed to stretching of the amine N-H group, which is caused by molecular hydrogen bonding in polymeric compounds, such as alcohols, phenols, and carboxylic acids. The peak at 2905 cm^−1^ was associated with C–H stretching vibrations, indicating the presence of lipid and phospholipid fractions [47]. The FTIR spectrum of the biomass sample showed a peak at 1624 cm^−1^, which corresponded to the stretching vibrations of the carbonyl (C=O) group or possibly the C=C stretching vibrations. The peak at 1406 cm^−1^ was strongly associated with carbonate minerals linked to the O-H group. In addition, the peak at 770 cm^−1^ was related to aromatic CH bending [48]. The FTIR spectra for biological synthesis revealed absorption peaks with a significant upward shift of the peak at 3428 cm^–1^ to a sharp peak at 3472 cm^–1^. FTIR spectra revealed new peak positions with notable morphological alterations compared with those of the control, where the peak at 2777 cm^–1^ in the phyto-AgNPs was due to the presence of an aldehyde C-H stretch and alkynes C≡C stretch [49]. A distinct peak at 1633 cm^–1^ is due to the amide C=O carbonyl stretch group, and the absorption peak at 1311 cm^–1^ is characteristic of nitro compound NO_2_ stretching [50]. The peak at 709 cm^−1^ corresponds to the bending of aromatic CH bonds. In addition, functional groups such as alkanes and aldehydes play crucial roles in metal absorption, confirming that the biologically synthesized AgNPs were coated with biological molecules.

Selecting reducing agents used during the synthesis process influenced the size and shape of the biosynthesized AgNPs. For example, Wei *et al*. [51] biosynthesized AgNPs using Chinese herbal medicine extracts, resulting in an average diameter of 10–20 nm.

In this study, TEM examination confirmed that the phyto-AgNPs prepared using *A. annua* extract were spherical and capped with a phytocompound. The TEM micrograph showed the surface of treated bacterial cells of the new clinical MRSA RM-Ph8 strain and revealed that the phytosynthesis had smaller spherical AgNPs than the chemical syntheses. Consequently, our phyto-AgNPs exhibited bactericidal action at a MIC of 50 mg/mL by inhibiting bacterial proliferation and achieving bacterial destruction within 24 h, including Gram-positive bacteria such as MRSA RM-Ph8, which have a thicker cell wall consisting of numerous layers of peptidoglycan and teichoic acids. Shaaban *et al*. [52] described a concept in which green NPs effectively disturbed the cell walls of *S. aureus* and *P. aeruginosa*, thereby demonstrating their efficiency, which is in agreement with the findings of Slavin *et al*. [53].

Sequencing of the 16S rRNA gene is widely used to identify microbes rapidly and easily. This technique also reveals how these organisms are related to each other and how they have evolved [54]. Identification of the new strain confirmed that the new clinical MRSA RM-Ph8 strain can be classified as bacteria, *Bacillota*, *Bacilli*, *Bacillales*, *Staphylococcaceae*, *and S. aureus* according to Bergeyʼs Manual of Systematic Bacteriology [55]. The phylogenetic tree showed a close genetic relationship with a <0.009 genetic relationship, which strongly confirmed its identity as *S. aureus*.

A biofilm consists of a community of bacterial cells that adhere to either living or non-living surfaces and are encased in a hydrated matrix of extracellular polymeric substances [56]. Harika *et al*. [29] reported that biofilms with tightly packed cells are breeding grounds for multidrug resistance. This occurs because plasmids harboring resistance genes can be easily exchanged between cells that are in close contact. Consequently, the bacteria within the biofilm matrix exhibit significantly greater resistance to antimicrobial agents.

The crystal violet assay employs a simple protein-dye that binds to molecules with negative charges on their surface, such as peptidoglycan and extracellular polysaccharide matrixes [57]. Crystal violet staining (1%) of the tissue culture plate confirmed that the new clinical strain formed a strong biofilm and treatment for 20 min with phyto-AgNPs at a concentration of 0.5 mg/mL decreased biofilm formation by 88%. These results demonstrate that the phyto-AgNP-induced detachment of the new clinical MRSA RM-Ph8 biofilm formation was rapid, effective, and occurred at AgNP concentrations that are achievable in clinical settings. In addition, the maximum bactericidal effect of these smaller phyto-AgNPs against bacterial strains was greater than that of larger spherical AgNPs [58].

TEM magnified the view of the cell envelope of the new strain *S. aureus* RM-Ph8 and focused on the effects of the new biogenic NPs, revealing leakage of the cellular content, loss of cellular flagella, and formation of large aggregates.

Metal NPs have considerable antibacterial effects on the surfaces of microbial organisms [59, 52]. Furthermore, Rai *et al*. [60] demonstrated that metal ions released from NPs can disrupt bacterial cell walls. Alavi *et al*. [61] reported that different treatments with NPs can control MDR. In contrast to antibiotics, AgNPs can readily penetrate the cellular membrane. In addition, extracts from *Psidium guajava* and other plant leaves exhibit significant antimicrobial activity against various veterinary Staphylococcal strains, which is noteworthy considering their antioxidant capabilities, as reported by Medina *et al*. [62]. Furthermore, AgNPs synthesized using the extract of the herb *Swertia paniculata* as a stabilizing agent against various pathogens hold great promise for biomedical applications, as demonstrated by Ahluwalia *et al*. [63].

### Therapeutic approach *in vivo*

The combination of the antibacterial agent *A. annua* with AgNPs had a notable effect on lung tissue *in vivo* against MRSA, demonstrating that AgNPs are a promising strategy for combating antibiotic-resistant bacteria [17]. The results revealed that phyto-AgNP treatment and *S. aureus* infection affected liver and kidney function biomarkers in rats. The serum levels of AST and ALT indicate hepatic function, and their elevation correlates with hepatic injury [64]. Infection with *S. aureus* leads to hepatic injury characterized by disruption of the stability and metabolism of hepatocyte membranes, resulting in changes in the serum levels of these enzymes. Elevated serum ALT and AST levels suggest hepatocyte dysfunction [65].

Consistent with the elevated ALT levels, ischemic regions with necrotic hepatocytes indicate *S. aureus* infection, as reported by Kolaczkowska *et al*. [66]. Furthermore, increased levels of liver enzymes are associated with the breakdown of liver cell membranes and exposure of cell contents [67]. However, according to Hassanen *et al*. [68], no notable differences were observed in the biochemical outcomes between the control and AgNP-treated groups.

The concentrations of ALT and AST, which are biomarkers of hepatic cell necrosis, were similar between the treatment and control groups. Therefore, the AgNPs developed in this study had no adverse effects on liver function. These results are consistent with those reported by Vasquez *et al*. [69], who observed that the administration of AgNPs to male rats did not result in fatalities, abnormal reactions, or significant toxic effects on liver indexes, indicating minimal NP toxicity. Furthermore, in the same study, pretreatment with AgNPs reduced liver cell damage. Consequently, AgNPs produced through green synthesis provide an economical and non-toxic technology that ensures the effectiveness and safety of their use in various scientific fields [70]. The current study agrees with the findings of Chatterjee *et al*. [71], who reported elevated blood ALT and AST levels, indicating liver injury.

Nonetheless, the decrease in serum transaminase (ALT and AST) levels relative to normal levels after treatment with AgNPs indicates the potential regeneration of hepatocytes and therapeutic effect on hepatic tissue. Histopathological findings in the liver of Group D revealed vacuolar degeneration and necrosis in numerous hepatocytes, areas of focal necrosis replaced by inflammatory cell aggregation and large areas of hemorrhage. The portal areas exhibited infiltrating mononuclear inflammatory cells alongside the congestion of portal blood vessels. These findings are consistent with those of Shamseldean *et al*. [72]. Immunohistochemical findings from Group D revealed robust expression of TNF-α and iNOS. TNF-α serves as a proinflammatory indicator [73], whereas iNOS acts as a proinflammatory and oxidative stress marker [74], and these proinflammatory cytokines attract inflammatory cells to the site of infection [75]. The correlation between histopathological alterations in infected tissue and cytokine expression indicates the involvement of cytokines in this mechanism. The expression of cytokines within tissues is associated with the migration of leukocytes into affected tissues and subsequent progressive tissue deterioration [76]. Groups E and F showed improvements in the histopathological lesions and immune histochemical expression of TNF-α and iNOS.

Reducing the production of free radicals or inducing antioxidant effects are effective methods for managing liver disease [77]. Given the potential toxicity of certain medications, there is a growing demand for alternative therapies, such as antioxidant-rich herbal medicines [78, 79]. Numerous plant-based remedies are used worldwide to treat liver ailments and improve liver function [80]. The use of plants to treat liver injuries is attributed to the presence of antioxidants as supplementary agents. Biosynthesized AgNPs are recognized for their antioxidant and free radical scavenging capabilities [81–83]. Thus, AgNPs enhance antioxidant capacity or exert notable effects on the restoration of the antioxidant system by altering enzymatic processes. This could be attributed to either the inhibition of free radical release or their protective effect against formation.

The leakage of cytoplasmic enzymes into the bloodstream following liver injury has harmful effects [84]. Therefore, increased cell membrane permeability and enzyme activity contribute to structural damage of the liver [85]. Increased concentrations of liver enzymes in the bloodstream originate from the release of serum enzymes due to lipid peroxidation [86]. In this study, serum enzyme levels, including AST and ALT, were elevated, indicating hepatic injury. The decrease in serum transaminase levels to nearly normal levels following treatment with biogenic AgNPs (Group F) suggests the potential for hepatocyte regeneration and therapeutic effects on liver tissue. Therefore, the antioxidant and free radical-scavenging properties of AgNPs may be critical for restoring the biochemical and histological parameters to near-normal levels in treatment groups [86].

## Conclusion

In this study, we successfully synthesized green AgNPs using a phytosynthesis method, thereby eliminating the need for chemicals, as confirmed by FTIR, TEM, and SEM analyses. A new clinical MRSA RM-Ph8 strain (accession number: OQ421819) was identified, which has a strong ability to form a biofilm at 0.008 mg/mL. The MIC of green AgNPs against the new clinical strain *Staphylococcus* RM-Ph8 was 50 mg/mL after 24 h of exposure. Phytochemical products with antibacterial properties may be viable alternatives to antibiotic-based growth promoters as feed additives for infected experimental animals. The protective effects of phyto-AgNP treatment on liver and kidney functions and tissues were revealed after 2 weeks in an *in vivo* experimental model. This highlights the potential of green AgNPs as a satisfactory therapeutic approach to combat the increasingly prevalent MDR human and veterinary pathogens, showing promise as a superior alternative to conventional antibiotics, which are increasingly ineffective against MDR bacteria.

## Authors’ Contributions

MTS: Conceptualization, data analysis, and validation. SHO: Conceptualization and experimental design of the animal model, statistical analyses, and reviewed the manuscript. MSA and FMA: Conceived and designed the analysis of microbial identification, NPs synthesis, their characterizations and interpretation of results, and manuscript preparation. RMSK: Conceptualization and experimental design for histopathology and experimental design. RHE: Data collection, methodology, and drafted the manuscript. All authors have read, reviewed, and agreed to the published version of the manuscript.
